# A comparison between rTMS and antidepressant medication on depressive symptom clusters in treatment-resistant depression

**DOI:** 10.1007/s00406-025-02012-0

**Published:** 2025-04-23

**Authors:** Iris Dalhuisen, Tom Biemans, Cornelis F. Vos, Sophie ter Hark, Iris van Oostrom, Jan Spijker, Ben Wijnen, Eric van Exel, Hans van Mierlo, Dieuwertje de Waardt, Martijn Arns, Indira Tendolkar, Joost Janzing, Philip van Eijndhoven

**Affiliations:** 1 Department of Psychiatry, Radboud University Medical Center, Nijmegen, The Netherlands; 2 Donders Institute for Brain Cognition and Behavior, Centre for Medical Neuroscience, Nijmegen, The Netherlands; 3 Antes, Parnassia Group, Rotterdam, The Netherlands; 4 Neurocare Clinics, Nijmegen, The Netherlands; 5 Depression Expertise Centre, Pro Persona Mental Health Care, Nijmegen, The Netherlands; 6 Behavioral Science Institute, Radboud University, Nijmegen, The Netherlands; 7 Center for Economic Evaluations, Trimbos Institute - Netherlands Institute of Mental Health and Addiction, Utrecht, The Netherlands; 8 Department of Psychiatry, GGZ inGeest Specialized Mental Health Care, Amsterdam, Netherlands; 9 Department of Psychiatry & Psychology, St. Antonius Hospital, Utrecht, Nieuwegein The Netherlands; 10 Department of Psychiatry, ETZ Hospital (Elisabeth-TweeSteden Ziekenhuis), Tilburg, The Netherlands; 11 Research Institute Brainclinics, Brainclinics Foundation, Nijmegen, The Netherlands; 12 Stanford Brain Stimulation Lab, Stanford University, Palo Alto, USA

**Keywords:** rTMS, Depression, Symptom clusters, Antidepressants, Treatment resistance

## Abstract

**Background:**

Antidepressive treatment outcomes can be assessed using sum scores from measurement scales, but symptom clusters may better capture the multidimensional structure of depression. Little is known about the comparative effectiveness of different treatment modalities on these clusters. We sought to evaluate the effects of rTMS and antidepressant medication on four symptom clusters and the extent to which these differ between treatments. In addition, we assessed whether baseline cluster scores predicted (non)response.

**Methods:**

Data were obtained from two clinical trials (DETECT: rTMS vs. medication; PITA: tricyclic antidepressants). Primary outcomes were symptom cluster scores: ‘General Depression’, ‘Anxiety’, ‘Somatic Symptoms’ and ‘Insomnia’. In the primary analysis based on DETECT, a MANCOVA comparing rTMS with medication was performed. For validation, a MANCOVA was performed replacing medication data from DETECT with data from PITA. Baseline symptom cluster scores were compared between responders and non-responders, as well as treatment groups.

**Results:**

In both the primary and validation analyses, no difference was seen between rTMS and medication on the symptom clusters. Similar patterns of response were observed in all groups, with ‘Insomnia’ showing the greatest effect of treatment. Baseline cluster scores did not predict treatment response.

**Conclusions:**

We did not find a differential effect of rTMS or antidepressant medication on depressive symptom clusters. Both treatments demonstrated comparable response patterns for all clusters, and baseline cluster scores did not differ between responders and non-responders. Future studies with larger samples or more homogeneous treatments may elucidate the role of symptom clusters as a tool for more individualized treatment.

**Trial registration:**

PITA NCT03548675 DETECT The Netherlands Trial Register NL7628.

**Supplementary Information:**

The online version contains supplementary material available at 10.1007/s00406-025-02012-0.

## Introduction

With over three hundred million people affected worldwide, Major Depressive Disorder (MDD) is one of the most common psychiatric disorders, as well as the second leading cause of disability [1, 2]. First-line treatments consist of pharmacotherapy, psychotherapy, or a combination of these [3]. Up to 35% of patients fail to respond to two trials of first-line treatments and are considered to have treatment-resistant depression (TRD) [4]. It is likely that symptom heterogeneity in depression, among others, is a contributing factor to this high rate of TRD, whereas parsing this heterogeneity may lead to more successful treatment strategies [5]. The diagnosis of MDD is commonly based on the Diagnostic and Statistical Manual of Mental Disorders (DSM-5), where the presence of at least five out of nine symptoms is required, of which at least one of the two core symptoms of depressed mood and anhedonia [6]. This results in a large number of possible symptom combinations, resulting in the symptom heterogeneity seen in patients sharing the same diagnosis. Not only do patients show heterogeneity in their symptom profiles, the recommended treatment options also vary widely in terms of their mechanism of action. For example, tricyclic antidepressants (TCAs) block the reuptake of norepinephrine and serotonin, monoamine oxidase inhibitors (MAIOs) inhibit the degradation of norepinephrine, serotonin and dopamine, and non-invasive brain stimulation (NIBS) methods such as electroconvulsive therapy (ECT) and repetitive transcranial magnetic stimulation (rTMS) modulate the brain by means of electrical stimulation through electrodes placed on the scalp or by focally stimulating cortical regions using electromagnetic pulses [7–9].

Although there are several modalities in the treatment guidelines, antidepressant treatment recommendations tend to focus more on initiating pharmacotherapy, and then switching, augmenting or using combination strategies, depending on the patient’s response to the earlier pharmacological treatments [10]. Knowledge about the matching of the appropriate therapeutic intervention(s) to the right patient based on individual characteristics, thereby potentially improving treatment response, is currently unavailable [11].

Depressive symptoms and treatment effectiveness are most commonly assessed with a validated clinician-administered assessment instrument, of which the Hamilton Depression Rating Scale (HDRS) is one of the most frequently used [12]. Although treatment effectiveness is typically measured by using the sum score, this approach has also been criticized [13]. When assessing treatment response based on a sum score, differential responses of unidimensional structures such as symptom clusters can go undetected [14].

In earlier work, efforts have been made to identify unidimensional clusters or subscales of items of the HDRS [15, 16]. The most comprehensive meta-analysis of the HDRS-17 factor structure known to date has been done by Shafer [17], who identified four depressive symptom clusters based on factor analysis (General depression, Anxiety, Insomnia and Somatic Symptoms) that also appeared to be the most generalizable to factors that were found in the majority of studies included in this analysis. This model extends the original three factor solution (General Depression or Symptom Severity, Agitation versus Retardation, and Insomnia) proposed by Hamilton with one factor [18, 19].

Different patterns of response have been reported for symptom clusters in both pharmacological and NIBS trials, with core mood showing a greater response to ECT than other symptom clusters [20]. Other examples include dysphoric symptoms showing a greater response to escitalopram than transcranial direct current stimulation (TDCS) whereas the opposite is true for sleep problems [21], and anxiosomatic symptoms showing the least response to rTMS [22]. These findings provide initial evidence for using symptom clusters as a clinical tool for a more personalized approach to antidepressant treatment. However, to the best of our knowledge, there is yet to exist a head-to-head comparison of the effectiveness of different treatment modalities on depressive symptom clusters. Furthermore, baseline scores of depressive symptoms have been shown to differ between responder and non-responders to a certain treatment, e.g. higher baseline anhedonia scores have been observed in non-responders to rTMS [23] and depressed patients that score high on anxiety respond better to ketamine than those with non-anxious depression [24].

To elaborate on this prior work, we sought to evaluate the effect of rTMS and antidepressant medication on the four predefined depressive symptom clusters as proposed by Shafer [17], and the extent to which this effect differs between the two treatments. Our primary analysis will be based on data from a randomized clinical trial (RCT) comparing rTMS and antidepressant medication, where medication could consist of TCAs or augmentation steps [25]. As an additional analysis to validate our results, the rTMS data from this RCT will also be compared with TCA data from an independent RCT with a larger sample size and more homogeneous treatment group [26]. Finally, we assessed whether baseline depressive symptom cluster scores are predictive of (non)response to rTMS or antidepressant medication.

## Materials and methods

### Study procedures

This work was a secondary analysis using data from two multicenter RCTs; DETECT and PITA. Full details of the study procedures for both studies can be found in the original publications [25–27]. Briefly, the DETECT study compared the effectiveness of rTMS with the next step in the medication algorithm of the Dutch treatment guidelines, which consisted of switching from the current antidepressant medication to a TCA or augmentation with lithium or a second-generation antipsychotic [28]. Our primary analysis compared the rTMS and medication groups of DETECT. Due to the heterogeneous nature of the medication group in this sample, a sensitivity analysis was performed excluding patients not treated with a TCA. For the validation analysis, the rTMS group was compared to data from the PITA study, as this is a larger and more homogeneous medication group. The PITA study evaluated pharmacogenetics-based dosing of TCAs, by directly comparing genotype-based dosing to dosing as usual. These two groups from PITA were combined for the current analysis.

### Participants

For a complete overview of all in- and exclusion criteria of both RCTs, see table S1 in the supplementary material. Only patients with HDRS-17 scores available for both baseline and end of treatment were included in the current analysis. Local research ethics board approval was obtained and all participants provided written, informed consent.

#### DETECT

Participants were outpatients above the age of 18 with a diagnosis of unipolar MDD confirmed using the Structured Clinical Interview for DMS-5 disorders I (SCID-I; 2018). Participants met the following inclusion criteria: current major depressive episode scoring > 16 on the HDRS-17, lack of response to at least two adequate treatment trials, and duration of the current episode less than two years.

#### PITA

Participants were in- and/or outpatients aged 18–65 years with a diagnosis of unipolar MDD confirmed using the SCID-I. Participants had a current major depressive episode scoring ≥ 19 on the HDRS-17 and had to be eligible for treatment with a TCA. Within the Dutch treatment guidelines TCAs are generally prescribed after inadequate response to (at least) a selective serotonine reuptake inhibitor (SSRI) [28].

### Treatment procedures

#### rTMS

The stimulation target (left dorsolateral prefrontal cortex; left DLPFC) was determined using the Beam F3 method [29]. rTMS was conducted using a high-frequency protocol (10 Hz), applying 60 trains with a duration of 5s and an inter-train interval of 25 s (50 pulses per train, 3000 pulses per session), at 120% resting motor threshold (rMT). Twenty-five rTMS sessions were given over the course of eight weeks, following a scheme of 4, 4, 4, 3, 3, 3, 2, 2 sessions per week.

### Medication

When discussing separate medication groups, they will be referred to as MED-DETECT and MED-PITA. Dosing of TCAs and lithium was done based on blood levels.

#### MED-DETECT

Treatment consisted of a switch from the current antidepressant medication to a TCA (nortriptyline, clomipramine and amitriptyline), or augmentation of the current antidepressant medication with lithium or a second generation antipsychotic, as prescribed in the Dutch treatment guidelines [28].

#### MED-PITA

Treatment consisted of TCAs (nortriptyline, clomipramine and imipramine) following the Dutch treatment guidelines and recommendations of the Dutch Pharmacogenomic Working Group [28, 30]. Patients randomized to dosing-as-usual started with the standard dose, whereas patients randomized to pharmacogenetics-based dosing had a start dose that was adjusted for their genotype.

### Outcome measures

Depressive symptom cluster scores based on the HDRS-17 were the primary outcomes. A total of 52 points can be scored on this scale, where nine items are scored between zero (not present) and four points (severe), while the remaining items are scored between zero (not present) and two points (severe). For both studies, the HDRS-17 was administered during screening, treatment and at the end of treatment (DETECT: after 8 weeks; PITA: after 7 weeks). The depressive symptom clusters used in this analysis were proposed by Shafer [17]. The four-component solution consists of the factors ‘General Depression’ (range: 0–20), ‘Anxiety’ (range: 0–18), ‘Somatic Symptoms’ (range: 0–8) and ‘Insomnia’ (range: 0–6). More information regarding the composition of these factors is displayed in table S2 in the supplementary material. To assess the level of treatment resistance in DETECT, the Dutch Measure for quantification of Treatment Resistance in Depression (DM-TRD) was used. Since the DM-TRD was not administered in PITA, the sum score of the pharmacotherapy items (6a and 6b) of the DM-TRD was determined based on pharmacological treatment history to compare the level of medication resistance between the two groups. Response based on the HDRS-17 is defined as a ≥ 50% reduction in total score.

### Statistical analyses

Statistical analyses were performed using SPSS Statistics 27.0 (IBM Corp., Armonk NY, USA) and procedures were 2-tailed with significance set at an alpha-level of 0.05, unless stated otherwise. T-tests and chi-squared tests were used to examine differences on demographic and clinical variables between the treatment groups. To check whether there was an effect of treatment on the four symptom clusters, paired samples t-tests were performed for rTMS, MED-DETECT and MED-PITA separately, for the pre- and post-treatment symptom cluster scores. We did not correct for multiple testing as the purpose of these analyses was solely to check for the presence of effects of these treatments on the clusters. In the primary analysis, to determine whether the effect of treatment on symptom clusters differed between rTMS and MED-DETECT, a MANCOVA was performed with age and gender as covariates, with symptom cluster change scores as dependent variable and treatment (rTMS/MED-DETECT) as between-subject factor. Baseline ‘Anxiety’ score was also added as a covariate, as the groups differed on this variable. As a sensitivity analysis, the MANCOVA was repeated with only the MED-DETECT patients that were treated with a TCA (TCA-DETECT). To validate the results of the primary analysis in a larger, independent medication sample, another MANCOVA was performed using the rTMS group of DETECT and MED-PITA, with the addition of DM-TRD med score, baseline ‘Anxiety’ score and baseline ‘Somatic Symptoms’ score as covariates, as the groups differed on these variables. In order to visualize comparisons of treatment effect between the symptom clusters for all three treatment groups, the sum score for each symptom profile was scaled using the proportion of maximum possible scaling method (POMS) [31]. A general linear model (GLM) with change in POMS score for each symptom cluster as within-subject factor and treatment group (rTMS/MED-DETECT/MED-PITA) as between-subject factor was performed to compare pattern of response between the groups and to compare changes in the four symptom clusters between themselves. Finally, to check whether baseline scores on the depressive symptom clusters differed between responders and non-responders, a logistic regression was performed with response (responders/non-responders) as dependent variable and baseline symptom cluster scores as independent variables. This regression was performed in the rTMS and MED-DETECT group separately. As a validation analysis, the model was performed in the MED-PITA group.

## Results

### Baseline characteristics

From DETECT 76 participants (rTMS: *n* = 44, MED: *n* = 32) were included and from PITA data from 83 participants were included. Baseline demographic and clinical characteristics are shown in Table 1. With regard to the primary analysis, the MED-DETECT and rTMS groups differed significantly on baseline score for ‘Anxiety’, *p* =.029. With regard to the sensitivity analysis, no differences were observed between the rTMS and TCA-DETECT (*n* = 17) groups (see supplementary table S3). For the validation analysis, the MED-PITA and rTMS groups differed significantly on DM-TRD medication score, indicating a lower level of medication resistance in the MED-PITA group (*p* <.001), baseline ‘Insomnia’ score (*p* =.006) and baseline ‘Somatic Symptoms’ score (p =.016). To check whether there was a treatment effect, paired samples t-tests were conducted for each symptom profile, for rTMS, MED-DETECT, and MED-PITA separately. For all three groups, there was a significant change in scores on all symptom clusters (see Table 2).

**Table 1:** Baseline characteristics for the rTMS, MED-DETECT and MED-PITA groups separately

	MED-DETECT (*n* = 32)	rTMS (*n* = 44)	*p*	MED-PITA (*n* = 83)	rTMS (*n* = 44)	*p*
Age (years)	40.3 ± 12.9	44.3 ± 14.9	0.233	41.5 ± 13.5	44.3 ± 14.9	0.302
Gender (m/f)	10/22	17/27	0.507	36/47	17/27	0.606
DM-TRD	10.2 ± 1.9	10.7 ± 2.7	0.313			
DM-TRD med				1.3 ± 0.5	1.9 ± 1.1	< 0.001*
HDRS-17 total	21.1 ± 4.5	21.8 ± 4.1	0.508	20.8 ± 4.7	21.8 ± 4.1	0.235
General Depression	9.0 ± 2.2	8.8 ± 2.0	0.561	8.3 ± 2.7	8.8 ± 2.0	0.303
Insomnia	2.6 ± 1.7	2.5 ± 1.7	0.966	3.4 ± 1.6	2.5 ± 1.7	0.006*
Anxiety	4.7 ± 2.3	5.9 ± 2.5	0.029*	5.2 ± 2.4	5.9 ± 2.5	0.137
Somatic symptoms	4.8 ± 1.6	4.6 ± 1.5	0.448	3.9 ± 1.6	4.6 ± 1.5	0.016*

Values represent mean ± SD or *N*. DM-TRD; Dutch Measure for quantification of Treatment Resistance in Depression; DM-TRD med: DM-TRD sum of pharmacotherapy items (6a and 6b); HDRS: Hamilton Depression Rating Scale. * indicates a statistically significant result

### Primary analysis

Overall treatment effect, based on total HDRS score, was greater for rTMS than for MED-DETECT (41.0% and 18.8% response, respectively). See also supplementary table S4). The MANCOVA comparing rTMS and MED-DETECT revealed no effect of treatment on the symptom clusters (Pillai’s trace, V = 0.080, F(4, 68) = 1.468, *p* =.221). As a sensitivity analysis the MANCOVA was rerun with the TCA-DETECT sample (*n* = 17). This also did not result in a significant effect (Pillai’s trace, V = 0.084, F(4, 54) = 1.235, *p* =.307).

**Table 2:** Change scores and results of paired samples t-tests for each symptom profile in the rTMS, MED-DETECT and MED-PITA groups separately

rTMS	Change score	*t*	*df*	*p*
General Depression	-4.0 ± 3.7	7.046	43	< 0.001*
Insomnia	-1.5 ± 1.8	5.418	43	< 0.001*
Anxiety	-3.0 ± 3.3	5.956	43	< 0.001*
Somatic symptoms	-1.5 ± 1.8	5.220	43	< 0.001*

MED-DETECT	Change score	*t*	*df*	*p*
General Depression	-1.8 ± 3.7	2.701	31	0.011*
Insomnia	-0.8 ± 1.6	2.726	31	0.010*
Anxiety	-1.0 ± 2.7	2.053	31	0.049*
Somatic symptoms	-0.7 ± 1.8	2.118	31	0.042*

MED-PITA	Change score	*t*	*df*	*p*
General Depression	-3.0 ± 3.1	8.728	82	< 0.001*
Insomnia	-1.1 ± 2.0	5.160	82	< 0.001*
Anxiety	-1.7 ± 2.8	5.457	82	< 0.001*
Somatic symptoms	-1.0 ± 1.8	4.814	82	< 0.001*

Values represent mean ± SD. * indicates a statistically significant result

### Validation analysis

Overall treatment effect was greater for rTMS than for MED-PITA (see supplementary table S4). The MANCOVA comparing rTMS and MED-PITA did not reveal a significant effect of treatment (Pillai’s trace, V = 0.053, F(4, 112) = 1.556, *p* =.191).

### Pattern of response

**Fig. 1:**
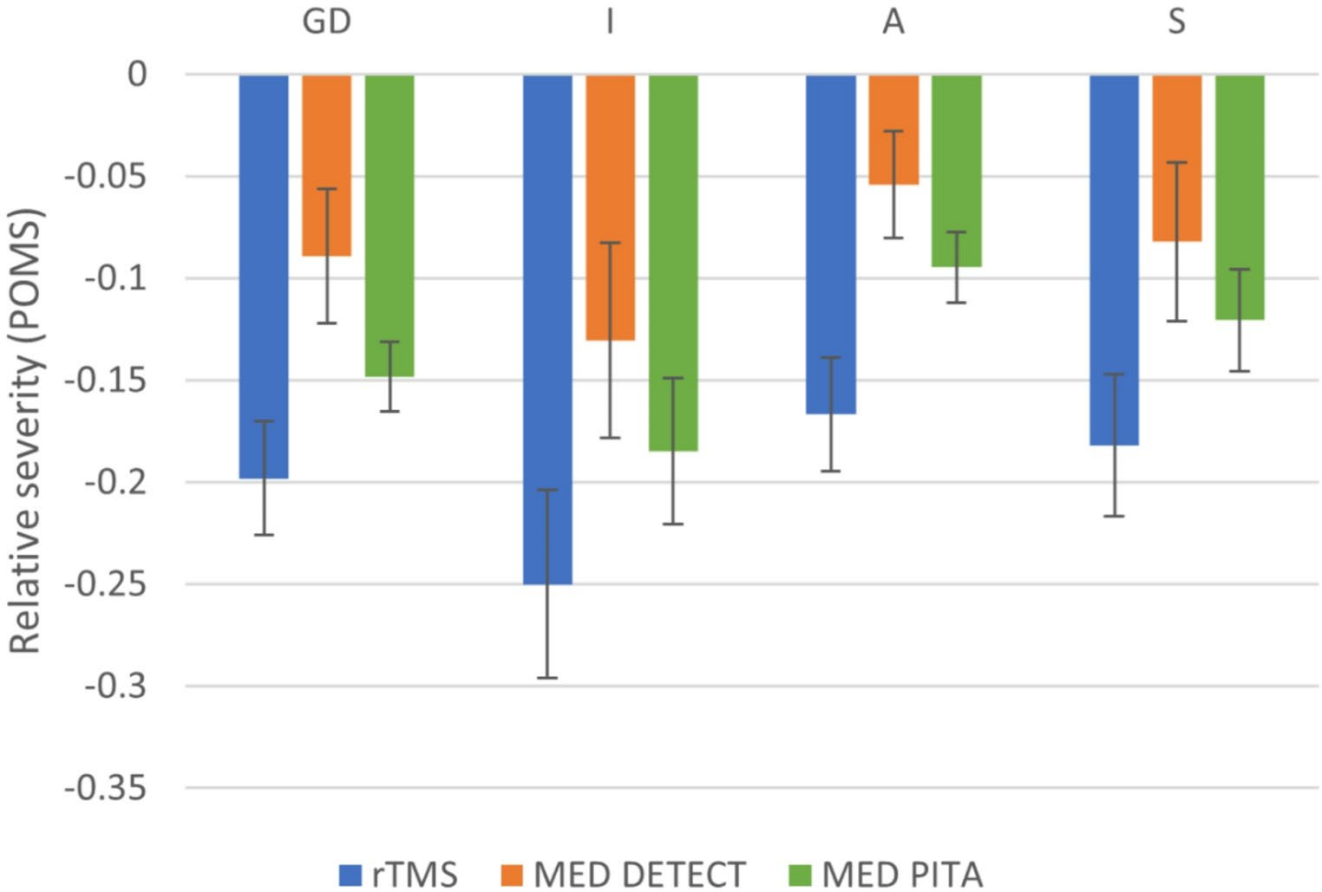
Bar graphs of change in relative severity of symptom clusters for rTMS, MED-DETECT and MED-PITA. Relative severity is based on the proportion of maximum scaling method (POMS) to facilitate comparisons between symptom clusters. GD: General Depression; I: Insomnia; A: Anxiety; S: Somatic Symptoms

See Fig. 1 for the relative change in symptom profile scores for the rTMS, MED-DETECT and MED-PITA groups. The GLM did not reveal any differential pattern of response between treatment groups (F(6, 468) = 0.054, *p* =.999), although contrasts between symptom clusters showed that relative change in symptom profile score was significantly greater for ‘Insomnia’ compared to ‘Anxiety’ (F(1, 156) = 8.243, *p* =.005).

### Predictor analysis

**Fig. 2:**
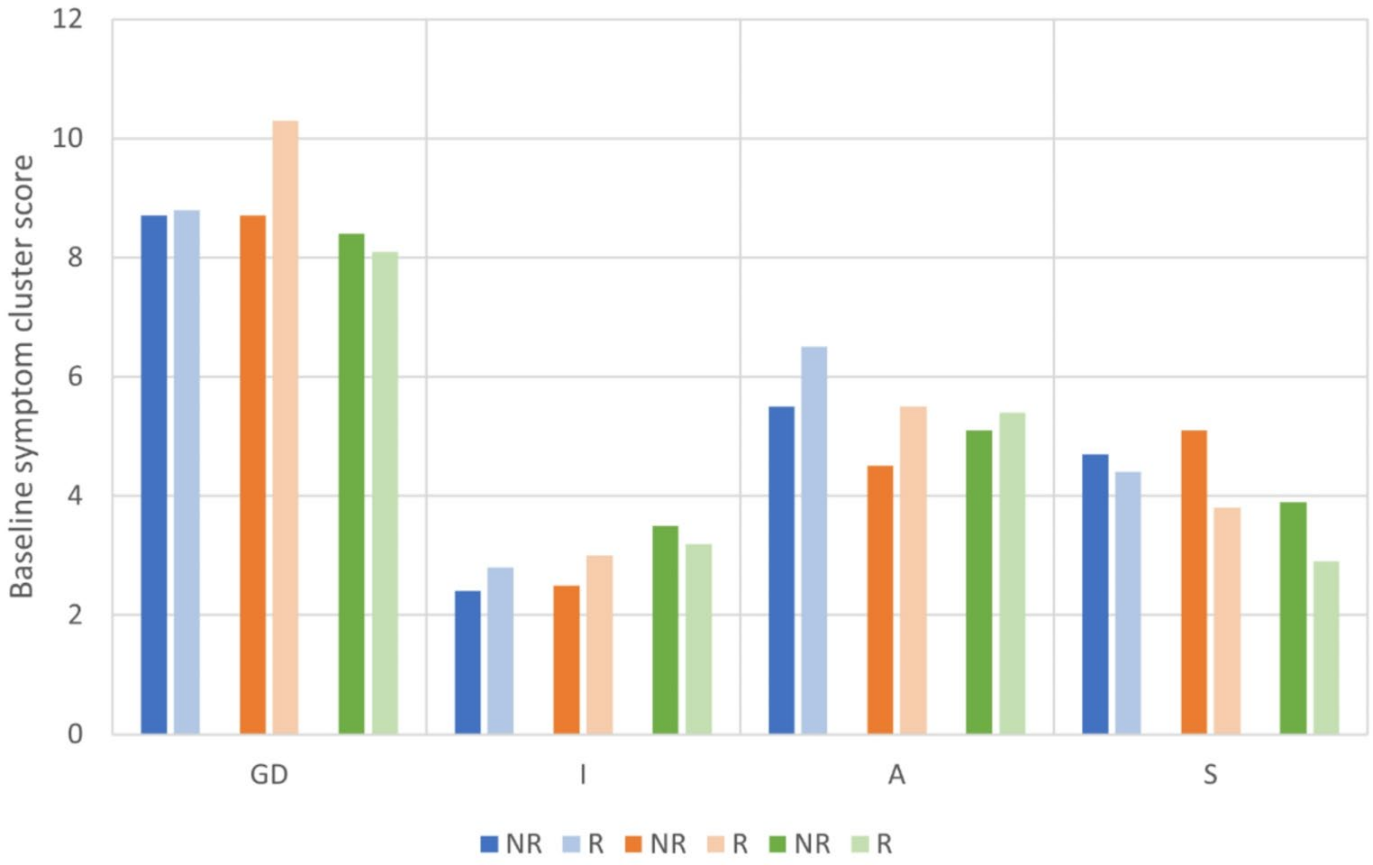
Bar graphs of baseline symptom cluster scores for rTMS (blue), MED-DETECT (orange) and MED-PITA (green). Bars are shown for non-responders and responders. GD: General Depression; I: Insomnia; A: Anxiety; S: Somatic Symptoms; NR: non-responders; R: responders

The logistic regression on depressive symptom clusters as predictor of response was not significant for rTMS. For MED-DETECT the model was significant (χ2(4) = 9.808, *p* =.044). Baseline somatic symptom cluster score significantly predicted (non-)response (B = -0.915, *p* =.040). The validation analysis in the MED-PITA group did not show a significant effect. See Fig. 2 for baseline symptom cluster scores for responders and non-responders in each group. See supplementary table S5 for descriptive statistics for responders and non-responders for each depressive symptom profile and in each group separately. Supplementary table S6 provides the model summaries of the logistic regressions.

## Discussion

In the first study on this subject we examined the effect of rTMS and antidepressant medication on depressive symptom clusters and whether this effect differed between these two treatment options. We performed a primary analysis based on data from the DETECT trial [25]. Because the antidepressant medication treatment options were varied in this study, a sensitivity analysis was performed using only TCA data from the DETECT trial. In addition, we performed a validation analysis in which the medication group from DETECT was replaced by a larger sample from the PITA study [26]. Furthermore, we examined whether baseline symptom profile score could be predictive for (non)response status. Treatment with both rTMS and antidepressant medication reduced scores on all four symptom clusters, although no differential effect between the treatment groups was observed in the primary, sensitivity, or validation analyses. The relative pattern of response also did not differ between the three groups, although overall a significantly greater relative change was observed for ‘Insomnia’ than for ‘Anxiety’. Finally, baseline depressive symptom profile scores did not predict treatment (non)response in the rTMS group. In the medication group from DETECT, the somatic symptom cluster score at baseline predicted treatment response, with a higher score indicative of a lower chance of response. The validation analysis in the PITA sample could not reproduce this effect.

In our primary, sensitivity, and validation analyses we did not find a differential effect of rTMS and antidepressant medication on the four depressive symptom clusters. To our knowledge, only one other study has compared depressive symptom clusters between two different treatment modalities [21]. In this study, patients were randomized to treatment with TDCS, escitalopram, or placebo. Escitalopram was more effective in reducing ‘core depressive’ and ‘guilt/anxiety’ symptoms, whereas TDCS was more effective in reducing symptoms of ‘sleep/insomnia’. Our findings are not consistent with the results from this study, which may be due to the differences in treatment or the smaller sample size in our study. Based on our study, we found no support for symptom cluster scores as a tool to reduce heterogeneity in depression based on an underlying neurobiological substrate on which medication and rTMS might act differently. Assuming that rTMS and antidepressant medication have a different neurobiological mechanism of action, one might hypothesize that these different working mechanisms might have an effect on different symptoms, however, our results do not support this.

The same differential pattern of response across the four symptom clusters was observed in all three treatment groups, with a greater relative improvement in ‘Insomnia’ compared to ‘Anxiety’. With regard to rTMS, this differential response pattern is congruent with neuroimaging-based findings from previous work hypothesizing distinct treatment targets for dysphoric (DLPFC) and anxiosomatic (dorsomedial prefrontal cortex; DMPFC) symptoms of depression [32]. Furthermore, building on previous work [22], this is the first study to demonstrate that the differential response of symptom clusters is consistent across different treatment modalities. We observed greater relative improvement for ‘Insomnia’ than for ‘Anxiety’. Insomnia is closely tied to core neurobiological mechanisms of depression, such as monoamine dysregulation and HPA axis dysfunction [33], whereas symptoms of anxiety involve additional, distinct pathways [34]. Moreover, anxiety includes significant cognitive components that may not be fully addressed with biological treatments alone, whereas insomnia is a relatively simpler biological condition.

One explanation for the similar relative response patterns between treatments could be that the symptoms that make up these symptom clusters influence each other and the clusters are therefore not completely independent, which would be consistent with the network approach to psychopathology [35]. In the primary analysis of the DETECT trial questionnaires were analyzed that specifically focused on one symptom dimension [36]. Based on one of these questionnaires, the State-Trait Anxiety Inventory (STAI), a significant difference was observed between the effect of rTMS and medication on anxiety. Interestingly, this difference was not present in the current analysis on the ‘Anxiety’ symptom profile. Questionnaires that focus specifically on one symptom may be more sensitive to change than a symptom profile based on a subset of items.

Finally, we found an effect of baseline somatic symptom cluster score on response to medication, with higher scores associated with non-response. Previous studies have found an association between baseline symptom scores and treatment outcome, although the predictive value was limited [23, 24, 37]. One study examined whether baseline features could predict response to rTMS [23]. Non-responders to rTMS were found to have higher baseline anhedonia scores, but neither positive nor negative predictive values reached significance. Another study found that scores on the symptom profile of ‘interest-activity’ (consisting of symptoms of low interest, reduced activity, indecisiveness and anhedonia) predicted treatment outcome to antidepressant medication [37]. However, several antidepressants were included in this analysis and the ‘interest-activity’ score predicted worse outcome for all of these antidepressants. Finally, although this treatment was not part of the current analysis, a study of the effects of ketamine classified depressed patients at baseline as anxious or non-anxious based on the ‘Anxiety’ factor of the HDRS-17 [24]. A greater reduction in depressive symptoms as measured by the MADRS was observed in anxious compared to non-anxious patients, but this difference was not present when looking at change in HDRS-17 score. Furthermore, there was no significant difference in the number of responders or time to response between the two groups. The clinical relevance of these predictors remains inconclusive.

### Strengths and limitations

Our analysis is the first comparative study of the effectiveness of rTMS and antidepressant medication on symptom clusters of depression. In addition, we attempted to validate our results by including independent data on TCAs as a comparison for the rTMS arm and to determine whether baseline depressive symptom clusters have predictive value. One limitation is the differences in the RCTs from which our treatment groups were drawn. First, the inclusion criteria differed slightly between the trials, with the PITA study requiring participants to have a higher HDRS-17 score at baseline, and thus more severe depression, to be included. Second, based on the DM-TRD medication score, participants from the DETECT study had, on average, failed more medication trials. A final difference is that treatment with rTMS was highly consistent across participants, whereas participants in the medication groups were treated with different TCAs. As different TCAs are commonly prescribed when different symptoms are more prominent, e.g. clomipramine for depressed patients with comorbid anxiety symptoms, this created heterogeneity in these treatment groups that may have influenced the results. A second limitation is our relatively small sample size, which prevented us from performing our own confirmatory factor analysis (CFA) on the symptom clusters of depression as proposed by Shafer [17], which requires sample sizes of 300–500 individuals [38]. Third, we examined individual symptom clusters, whereas perhaps an overall profile based on different combinations of depressive symptom profile scores (i.e. high ‘General Depression’; high ‘Anxiety’; low ‘Insomnia’; low ‘Somatic Symptoms’) might provide a more comprehensive impression and thus be more sensitive to differential treatment effects.

### Future directions

This work builds on previous research exploring the potential of symptom clusters for a more personalized approach to treatment strategies, both within and between treatments. Although this has not been the focus of the current study, future work focusing on the personalization of rTMS could use symptom clusters to target patient-specific symptoms and adjust treatment targets, as suggested by Siddiqi and colleagues [32]. To explore the potential of symptom clusters as predictors of treatment response, combining data from both RCTs and clinical practice, as well as from different treatment modalities, could provide more knowledge about which intervention works best for a given baseline symptom profile, although this would remain observational. These results could then inform studies specifically investigating whether stratification based on symptom clusters leads to better treatment outcomes. Finally, it is worth investigating whether the use of specific questionnaires for each symptom profile would have added value over the HDRS-17 factor solution, as these questionnaires may be more sensitive to change, thereby increasing the chance of detecting differential response patterns [17].

## Conclusion

In patients with treatment-resistant depression, we did not find a differential effect of rTMS and antidepressant medication on depressive symptom clusters based on the four predetermined factors of the HDRS-17. Both treatments show the same relative pattern of response for the symptom clusters, with greater effect on symptoms of ‘Insomnia’ as opposed to ‘Anxiety’. The lack of effects in the current study may be due to small sample size or treatment heterogeneity. Future studies with larger samples or with more homogeneous treatments may show whether symptom clusters can be used as a tool for a more individualized approach to treatment and/or target selection, potentially increasing treatment effectiveness in TRD patients.

## Electronic supplementary material

Below is the link to the electronic supplementary material.

Supplementary Material 1
