# Supplementary material for: A comparison between rTMS and antidepressant medication on depressive symptom clusters in treatment-resistant depression
Source: Eur Arch Psychiatry Clin Neurosci. 2025 Apr 23;275(6):1799–807. doi: 10.1007/s00406-025-02012-0 (PMC12500816; doi:10.1007/s00406-025-02012-0)
Supplement: Supplementary file 1 — Supplementary Material 1 [file 406_2025_2012_MOESM1_ESM.docx]

Supplementary Material

A comparison between rTMS and antidepressant medication on depressive symptom clusters in treatment-resistant depression

Iris Dalhuisen^1†^*, Tom Biemans^1,2†^, Cornelis F. Vos^1,3^, Sophie ter Hark^1,2^, Iris van Oostrom^4^, Jan Spijker^5,6^, Ben Wijnen^7^, Eric van Exel^8^, Hans van Mierlo^9^, Dieuwertje de Waardt^10^, Martijn Arns^11,12^, IndiraTendolkar^1,2^, Joost Janzing^1^, & Philip van Eijndhoven^1,2^

*Table S1*. A complete overview of all in- and exclusion criteria for both studies.

| PITA | DETECT |
| --- | --- |
| Inclusion criteria | Inclusion criteria |
| Age between 18 and 65 years | Minimum age of 18 years |
| Severe depression (HDRS-17 > 19), without psychotic features | Moderate to severe depression (HDRS-17 > 16), without psychotic features |
| Patients included, who were, according to their physicians, eligible for TCA treatment (nortriptyline, clomipramine or imipramine) |  |
|  | Failed at least two adequate treatments trials, including at least one treatment with antidepressants |
|  | Duration of current depressive episode < two years |
| Exclusion criteria | Exclusion criteria |
| Diagnosis of bipolar disorder, schizophrenia, substance dependence | Diagnosis of bipolar disorder, schizophrenia, schizoaffective disorder, current substance dependence, or organic psychosyndrome |
| Pregnancy or breastfeeding | Pregnancy |
| Mental retardation (IQ < 80) |  |
| Medical contraindication for TCA use, such as recent myocardial infarction, other drugs influencing the pharmacokinetics of the TCAs as based on a list of interacting drugs (other psychotropic medication except for benzodiazepines up to 4mg dose equivalent) |  |
|  | The presence of a concurrent significant medical condition impeding the ability to participate |
|  | Prior treatment with rTMS or ECT |
|  | Epilepsy, convulsion or seizure |
|  | Serious head trauma, head surgery |
|  | Ferromagnetic metal parts in the head (except dental wire) |
|  | Implanted cardiac pacemaker or neurostimulator. |

*Table S2.* The four-component solution for the symptom profiles of depression as proposed by the meta-analysis by Shafer.

| Number | Item | General Depression | Anxiety | Somatic Symptoms | Insomnia |
| --- | --- | --- | --- | --- | --- |
|  |  |  |  |  |  |
| 1 | Depressed mood | 4 |  |  |  |
| 2 | Guilt | 4 |  |  |  |
| 3 | Suicide | 4 |  |  |  |
| 7 | Work and interests | 4 |  |  |  |
| 8 | Retardation | 4 |  |  |  |
| 9 | Agitation |  | 4 |  |  |
| 10 | Anxiety psychic |  | 4 |  |  |
| 11 | Anxiety somatic |  | 4 |  |  |
| 15 | Hypochondriasis |  | 4 |  |  |
| 17 | Insight loss |  | 2 |  |  |
| 12 | Gastrointestinal |  |  | 2 |  |
| 13 | General somatic |  |  | 2 |  |
| 14 | Libido loss |  |  | 2 |  |
| 16 | Weight loss |  |  | 2 |  |
| 4 | Insomnia initial |  |  |  | 2 |
| 5 | Insomnia middle |  |  |  | 2 |
| 6 | Insomnia delayed |  |  |  | 2 |
|  |  |  |  |  |  |
| Total Score symptom profile | | 20 | 18 | 8 | 6 |

*Table S3.* Baseline characteristics for the rTMS and patients from MED-DETECT treated with TCAs separately.

|  | TCA-DETECT (*n* = 17) | rTMS (*n* = 44) | *p* |
| --- | --- | --- | --- |
| Age (years) | 37.2 ± 11.2 | 44.3 ± 14.9 | .082 |
| Gender (m/f) | 5/12 | 17/27 | .501 |
| DM-TRD | 9.9 ± 2.2 | 10.7 ± 2.7 | .273 |
| HDRS-17 total | 21.4 ± 5.5 | 21.8 ± 4.1 | .759 |
| General Depression | 9.1 ± 2.5 | 8.8 ± 2.0 | .611 |
| Insomnia | 2.4 ± 1.9 | 2.5 ± 1.7 | .703 |
| Anxiety | 4.8 ± 2.6 | 5.9 ± 2.5 | .119 |
| Somatic symptoms | 5.2 ± 1.7 | 4.6 ± 1.5 | .180 |

Values represent mean ± SD or *N*. DM-TRD; Dutch Measure for quantification of Treatment Resistance in Depression; DM-TRD med: DM-TRD sum of pharmacotherapy items (6a and 6b); HDRS: Hamilton Depression Rating Scale.

*Table S4.* Response, remission and change score on the HDRS.

|  | Total sample (*n* = 76) | MED-DETECT (*n* = 32) | rTMS (*n* = 44) | MED-PITA  (*n* = 83) |
| --- | --- | --- | --- | --- |
| Response  Remission | 24 (31.6 %)  15 (19.7 %) | 6 (18.8 %)  2 (6.3 %) | 18 (41.0 %)  13 (29.5 %) | 23 (27.7%)  15 (18.7%) |
| HDRS-17 total change | -7.5 ± 8.0 | -4.2 ± 7.1 | -9.9 ± 7.7 | -6.7 ± 6.5 |

Values represent mean ± SD or *N* (%). HDRS: Hamilton Depression Rating Scale.

*Table S5*. Mean and standard deviations for responders and non-responders for each baseline depressive symptom profile score and in each treatment group separately.

|  | rTMS (*n* = 44) | | MED-DETECT (*n* = 32) | | MED-PITA (*n* = 83) | |
| --- | --- | --- | --- | --- | --- | --- |
|  | NR | R | NR | R | NR | R |
| General Depression | 8.7 ± 2.0 | 8.8 ± 2.0 | 8.7 ± 2.2 | 10.3 ± 2.1 | 8.4 ± 2.8 | 8.1 ± 2.4 |
| Insomnia | 2.4 ± 1.6 | 2.8 ± 1.9 | 2.5 ± 1.6 | 3.0 ± 2.4 | 3.5 ± 1.7 | 3.2 ± 1.4 |
| Anxiety | 5.5 ± 2.6 | 6.5 ± 2.2 | 4.5 ± 1.8 | 5.5 ± 3.9 | 5.1 ± 2.4 | 5.4 ± 2.5 |
| Somatic Symptoms | 4.7 ± 1.3 | 4.4 ± 1.8 | 5.1 ± 1.5 | 3.8 ± 1.8 | 3.9 ± 1.5 | 2.9 ±1.7 |

Values represent mean ± SD. R: response; NR: non-response.

*Table S6*. Logistic regression model summary.

|  | rTMS (*n* = 44) | | | MED-DETECT (*n* = 32) | | | MED-PITA (*n* = 83) | | |
| --- | --- | --- | --- | --- | --- | --- | --- | --- | --- |
|  | *B* | *SE* | *p* | *B* | *SE* | *p* | *B* | *SE* | *p* |
| General Depression | 0.033 | 0.175 | .850 | 0.586 | 0.328 | .074 | -0.054 | 0.096 | .572 |
| Insomnia | 0.100 | 0.195 | .609 | 0.433 | 0.346 | .211 | -0.102 | 0.160 | .522 |
| Anxiety | 0.173 | 0.138 | .212 | 0.148 | 0.261 | .571 | 0.051 | 0.105 | .626 |
| Somatic Symptoms | -0.097 | 0.228 | .669 | -0.915 | 0.446 | .040 | 0.032 | 0.165 | .845 |
